# MARKERS OF GLUCOSE METABOLISM ARE ASSOCIATED WITHCCOGNITIVE DECLINE AMONG FAMILIES WITH EXCEPTIONAL SURVIVAL

**DOI:** 10.1093/geroni/igad104.2951

**Published:** 2023-12-21

**Authors:** Iva Miljkovic, Ryan Cvejkus, Stacy Andersen, Stephanie Cosentino, Joseph Zmuda

**Affiliations:** University of Pittsburgh, Pittsburgh, Pennsylvania, United States; University of Pittsburgh, Pittsburgh, Pennsylvania, United States; Boston University Chobanian & Avedisian School of Medicine, Boston, Massachusetts, United States; Columbia University Medical Center, New York City, New York, United States; University of Pittsburgh, Pittsburgh, Pennsylvania, United States

## Abstract

Although the connection between type 2 diabetes (T2D) and dementia is known, it is less clear whether this relationship extends to cognitive decline. Our aim was to examine whether baseline fasting plasma glucose, insulin, HOMA-IR, and HbA1c, and T2D status predict cognitive decline in a multicenter cohort study of families with a clustering of exceptional survival. We analyzed change across five cognitive tests collected over 7.9 years: Mini-Mental State Examination, Digit Span, Logical Memory, Animal Fluency and Digit Symbol Substitution. Among 2,175 family members (mean age 63±11 years (range 32-100); 54% women), 39.8% had prediabetes and 6.8% had T2D. Multiple linear mixed models were adjusted for age, sex, field center, education, APOEε4 status, BMI and family relatedness. A trend was observed for immediate (p=0.047) and delayed (p=0.037) memory, with normal glucose tolerance (NGT) predicting increased scores, prediabetes showing no change, and T2D predicting decreased scores. Among those without T2D (N=2,026), each standard deviation (SD) higher glucose (11.5 mg/dl) was related to a greater decline in delayed memory (-0.34±0.16mg/dl; p=0.032), each SD higher HbA1c (0.32%) was related to a greater decline in immediate (-0.36±0.15 mg/dl; p=0.016) and delayed (-0.31±0.16 %; p=0.05) memory, while each SD higher HOMA-IR (1.5) was related to a greater decline in Digit Span Backwards (-0.13±0.06; p=0.047). Findings suggest that among individuals with a clustering of exceptional survival, T2D, glucose, and HbA1c may predict greater decline in episodic memory, an ability typically affected early in Alzheimer dementia. The possible mechanisms, including genetic and metabolic mediators, warrant further investigation.

